# Anx2 Interacts with HIV-1 Gag at Phosphatidylinositol (4,5) Bisphosphate-Containing Lipid Rafts and Increases Viral Production in 293T Cells

**DOI:** 10.1371/journal.pone.0005020

**Published:** 2009-03-27

**Authors:** Alexia V. Harrist, Elena V. Ryzhova, Thomas Harvey, Francisco González-Scarano

**Affiliations:** Departments of Neurology and Microbiology, University of Pennsylvania, Philadelphia, Pennsylvania, United States of America; University of California San Francisco, United States of America

## Abstract

The neuronal damage characteristic of HIV-1-mediated CNS diseases is inflicted by HIV-1 infected brain macrophages. Several steps of viral replication, including assembly and budding, differ between macrophages and T cells; it is likely that cell-specific host factors mediate these differences. We previously defined Annexin 2 (Anx2) as an HIV Gag binding partner in human monocyte-derived macrophages (MDMs) that promotes proper viral assembly. Anx2, a calcium-dependent membrane-binding protein that can aggregate phospholipid-containing lipid rafts, is expressed to high levels in macrophages, but not in T lymphocytes or the 293T cell line. Here, we use bimolecular fluorescence complementation in the 293T cell model to demonstrate that Anx2 and HIV-1 Gag interact at the phosphatidylinositol (4,5) bisphosphate-containing lipid raft membrane domains at which Gag mediates viral assembly. Furthermore, we demonstrate that Anx2 expression in 293T cells increases Gag processing and HIV-1 production. These data provide new evidence that Anx2, by interacting with Gag at the membranes that support viral assembly, functions in the late stages of HIV-1 replication.

## Introduction

Direct infection of the brain by HIV-1 causes the AIDS-definining illness HIV-associated dementia (HAD) as well as a spectrum of motor and cognitive disorders [1], [2]. Though neuronal death and damage are a hallmark of the pathology associated with HAD, neurons themselves are not infected. Instead, monocyte-derived macrophages (MDMs) and parenchymal microglia are the productively infected cells within the brain [2], [3], and it is the viral proteins and inflammatory mediators released by these cells that damage neurons either directly or by causing glial dysfunction (reviewed in [2], [4]. Moreover, as HIV enters the brain within weeks after initial infection and potentially years before neurological symptoms develop [5], [6], it is likely that brain macrophages serve as long-lived viral reservoirs in the immunologically and pharmacologically protected central nervous system (CNS) [7]. Given the critical role of macrophages in the neuropathogenesis of AIDS, understanding aspects of HIV replication that may be unique to these cells is a crucial step toward developing more specific and effective antiretroviral therapies to treat HIV-mediated CNS disease.

Several aspects of HIV replication differ in macrophages from the more extensively studied CD4+ T cells, including the coreceptor used for entry, the mechanisms regulating nuclear import and viral transcription, and the cellular location of assembly and budding (reviewed in [8]). As these cells serve vastly different functions in the immune response, it is likely that many of the differences in viral replication are due to differential protein expression and regulation between the two cell types. We recently undertook studies to identify macrophage-specific proteins that function in HIV-1 assembly and budding [9].

HIV-1 assembly is mediated by the viral polyprotein p55^Gag^, the only viral protein necessary for the formation and release of virion-like particles [10]. Gag is synthesized as a 55 kD precursor with four domains that mediate the steps of virion assembly, budding, and release [11]. During or immediately after assembly, the viral protease cleaves p55^Gag^ into the matrix (MA, p17), capsid (CA, p24), and nucleocapsid (NC, p7) proteins that form the structure of the mature, infectious virion.

Recent evidence has shown that Gag preferentially mediates viral assembly at specific membrane microdomains. These domains are cholesterol-enriched lipid rafts that contain the phospholipid phosphatidylinositol (4,5) bisphosphate (PtdIns(4,5)P_2_) [12]–[24] and that are enriched in endosomal tetraspannins and sorting machinery [25]–[27]. Host proteins that regulate membrane phospholipids, cholesterol, and endosomal components may thus play important roles in the late stages of viral replication. We identified Anx2 as a HIV-1 Gag-interacting protein in productively infected MDMs in an effort to discover cellular proteins that influence viral assembly and budding in these cells [9], [28]. Anx2 is expressed highly in macrophages but not in T cells [9], and the described functions of Anx2 make it a good candidate as a possible regulator protein.

Anx2 is a multifunctional calcium-dependent membrane-binding protein with roles in both extracellular signaling and the intracellular regulation of membrane composition and dynamics. Extracellular and cell-surface associated Anx2 is involved in plasmin generation [29] and cytokine production by macrophages [30], [31], and has been proposed to facilitate HIV-1 binding to and entry into macrophages [32]. In the cytoplasm, Anx2 binds to endosomal membranes [33] and is important in both early and late phases of the endocytic pathway [34]–[37]. The mechanisms by which Anx2 functions in these dynamic membrane events may involve one of several abilities to influence membrane composition and aggregation. Anx2 can mediate the lateral association and stability of cholesterol-rich microdomains [38]–[40], concentration of proteins destined for endocytic pathways [41]–[44] and induction of the formation of stable PtdIns(4,5)P_2_ clusters within membranes [45]. The ability of Anx2 to regulate endosomal trafficking as well as cholesterol and PtdIns(4,5)P_2_ within cellular membranes make it an attractive host protein important for HIV-1 assembly and budding in macrophages.

Our previous experiments [9] suggested a role for Anx2 in viral assembly based on the following evidence: *first*, Anx2 and Gag were only co-immunoprecipitated in activated, productively infected macrophages and not in quiescent macrophages in which there was a block to HIV replication at a late step in the viral life cycle [46]; *second*, both Gag and Anx2 colocated with CD63+ late endosomal compartments in infected macrophages, the same compartments in which mature virions accumulate [9], [47]–[50]; and *third*, downregulation of Anx2 resulted in decreased viral replication, aberrant Gag processing, and failure of mature virions to accumulate in intracellular vesicles. A subsequent study by Chertova et al. demonstrated that Anx2 is incorporated into virions released from HIV-1 infected MDMs [51], presenting further evidence that it is present at the site of viral assembly.

As MDMs are difficult to transfect and display donor-to-donor variability in protein expression, we chose here to use the 293T cell line to further define the role of Anx2 in viral replication. Multiple recent studies have taken advantage of the ease of transfection of epithelial cell lines such as 293T and HeLa cells to study the role of host components such as PtdIns(4,5)P_2_ [20], [22], cholesterol [16], [17], [19], [24], and host proteins [52]–[58] in HIV-1 assembly and budding. Furthermore, as 293T cells do not express endogenous Anx2, they allow us to control Anx2 expression levels and give us a model in which to study the effects of Anx2 gain-of-function on viral replication. Based on our previous observations in MDMs, we hypothesized that Anx2 helps form a membrane scaffold from which HIV-1 buds. Here, we investigate the location and nature of the membranes at which Anx2 and Gag interact and confirm these proteins bind at the PtdIns(4,5)P_2_–enriched lipid rafts at which HIV assembles. Furthermore, in accordance with our loss-of-function findings in MDMs, we report that Anx2 gain-of-function enhances Gag processing and viral production. These studies contribute to our understanding of HIV-1 replication in macrophages, the major mediators of HIV-1 associated neurologic disease.

## Results

### Anx2 was incorporated into virions produced by MDMs and 293T cells

**Figure 1:**
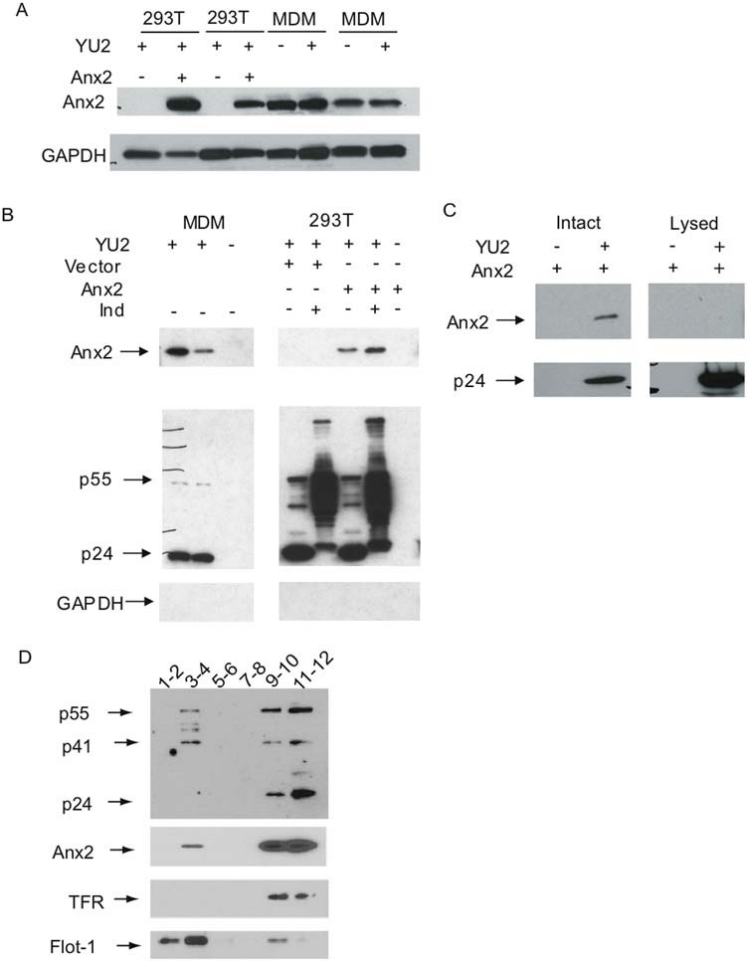
Anx2 was present in virions released from 293T cells and localized with Gag to detergent resistant membrane domains. (A) Western blot analysis of lysates from 293T cells transfected with HIV-1_YU2_+Vector or HIV-1_YU2_+Anx2 and MDMs either uninfected or infected with HIV-1_YU2_. Anx2 expression in transfected 293T cells was comparable to that in MDMs. (B) (Left Panel) Human peripheral blood mononuclear cells (PBMCs) were differentiated into monocyte-derived macrophages (MDMs) and infected for 48 hr with HIV-1_YU2_ (200 ng p24^Gag^/six-well plate well). The supernatants collected eleven days after infection were clarified, and aliquots of 1 mL were passed through 0.45 µM filters. Virus pellets were obtained by ultracentrifugation of the filtered supernatants and analyzed by SDS-PAGE and western blot. Anx2 was present in the pellets obtained from the supernatants of infected macrophages but not in those from uninfected macrophages. GAPDH was not detected, ensuring that the pellet was not contaminated with free cellular material. (Right Panel) 293T cells were cotransfected with plasmids encoding HIV-1_YU2_ and Anx2, HIV-1_YU2_ and empty vector, or Anx2 alone. As indicated, some cells were treated with 50 µM indinavir sulfate (IND – an HIV protease inhibitor) immediately after transfection. Thirty-six hours later, the supernatants were collected and virions were pelleted and analyzed in the same manner as those from the MDMs. Anx2 was also present in the pellets from 293T cells cotransfected with HIV-1_YU2_ and Anx2 but not under the control conditions. Indinavir sulfate had no effect on the apparent level of Anx2 in the virion pellet over several experiments. In the absence of protease inhibitor, the Gag signal had mobility consistent with mature capsid, but it was a larger smeared band in the presence of the inhibitor. (C) Supernatants from 293T cells transfected with Anx2 alone or with HIV-1_YU2_ and Anx2 were precleared with Protein G agarose beads before incubation with HIV-Ig to bind either intact virions or virions lysed in 0.5% Triton-X-100. Western blot analysis of immunoprecipitated proteins demonstrates that Anx2 was present with mature capsid (p24^Gag^) in intact virions. Lysing the virions prevented the co-immunoprecipitation of capsid and Anx2, indicating the Anx2-Gag interaction is lost in the mature virion. (D) 293T cells were cotransfected with plasmids encoding HIV-1_YU2_ and Anx2. Thirty-six hours later, cells were subjected to membrane flotation ultracentrifugation in the presence of 1% Triton X-100 as described in materials and methods. Fractions were combined, concentrated, and analyzed by SDS-PAGE and western blot against Gag, Anx2, the non-raft-associated membrane protein Transferrin receptor (TFR), and the raft-associated membrane protein Flotillin-1 (Flot-1). Detergent resistant membranes floated to fractions 2–5 while cytosol and detergent sensitive membrane remained in fractions 9–12. Anx2 and Gag were present in the lipid raft domains.

We previously reported that Anx2 and Gag co-precipitated when co-transfected into 293T cells [9]. To examine whether Anx2 and Gag interact at the site of viral assembly, we cotransfected 293T cells with a molecular clone expressing Anx2 and the infectious clone HIV-1_YU2_, an M-tropic provirus cloned from the brain of a patient with the AIDS dementia complex [59], [60]. Transient transfection of Anx2 into these cells resulted in Anx2 expression levels comparable to that in both mock- and HIV-infected macrophages with some experiment-to-experiment variability much like the donor-to-donor variability seen with primary macrophages **(** **Fig. 1A** **)**. Untransfected 293T cells expressed no detectable endogenous Anx2.

As an initial step in determining the site of interaction, we first examined whether Anx2 was present in released virions. Chertova et al. (2006) recently reported that Anx2 is incorporated into virions produced by HIV-1 infected MDMs, evidence that in these cells viral assembly occurs at membranes on which this protein is concentrated [49], [50]. To confirm this finding, supernatants from HIV-1_YU2_-infected MDMs were filtered through 0.45 um filters to remove cells, and viral particles were precipitated by ultracentrifugation before analysis by SDS-PAGE and western blot. As shown in **Fig 1B**, the pellets from infected macrophages contained Anx2 as well as mature capsid (p24^Gag^), confirming that Anx2 was indeed incorporated into virions. The cellular release of microvesicles containing Anx2 was an unlikely alternative explanation, as the pellets from uninfected MDMs contained no Anx2. The absence of cellular debris in the pellets was confirmed by the absence of GAPDH.

We next determined whether in 293T cells Anx2 was also present at the site of viral assembly. Virions in the supernatants of 293T cells cotransfected with HIV-1_YU2_ and Anx2, HIV-1_YU2_ and empty vector, or Anx2 alone were pelleted in the same way as the MDM virions. Anx2 was present in virions from 293T cells overexpressing the protein, but not in cells cotransfected with HIV-1_YU2_ and empty vector (**Fig. 1B**). To determine whether the extent of Gag processing influenced Anx2 incorporation, we also analyzed virion-like particles released from cells treated with the protease inhibitor indinavir sulfate (Ind) and in multiple repetitions of the experiment we did not observe significant differences in the extent of Anx2 incorporation. Anx2 was not present in pellets from 293T cells transfected with Anx2 alone, and GAPDH was absent from all pellets, again indicating that cellular microvesicles or secreted Anx2 were an unlikely source of the Anx2 in the pellets.

To confirm the direct association between HIV-1 virions and Anx2, we immunoprecipitated virions using HIV-IG™ (see Materials and Methods) and analyzed precipitated virions for the presence of Anx2 by SDS-PAGE and western blot. Virus precipitated in the absence of detergent from the supernatants of 293T cells cotransfected with HIV-1_YU2_ and Anx2 contained Anx2 **(** **Fig. 1C** **)**; the presence of Anx2 was not due to non-specific binding of HIV-IG to Anx2, as Anx2 was not brought down with this procedure from supernatants of 293T cells transfected with Anx2 alone. Interestingly, Anx2 was not pulled down when virions were lysed in 0.5% Triton X-100 prior to immunoprecipitation **(** **Fig. 1C** **)**, suggesting that while Anx2 initially binds Gag and is incorporated into virions during the process of viral assembly, the Gag-Anx2 association is lost within the infectious virion, perhaps due to the processing of full-length Gag. These data confirm that Anx2 was incorporated into HIV-1 released from 293T cells, as it is from HIV-1 released from macrophages, and thus provide evidence that Anx2 was present at the site of viral assembly in these cells.

### Anx2 and Gag were both present in detergent-resistant membrane fractions of 293T cells

Gag preferentially mediates HIV-1 assembly and budding from detergent-resistant, cholesterol-rich lipid raft membrane domains [15], [19], [24], [47], [61]. To determine whether Gag and Anx2 are both present in these membrane domains in HIV-1_YU2_ and Anx2 cotransfected 293T cells, we performed membrane flotation ultracentrifugation using a protocol previously employed to study the extent of Gag-membrane binding [19], [62]–[64]. Thirty-six hours after transfection, cells were lysed in buffer containing 1% Triton X-100, adjusted to 73% sucrose, overlayed with 65% and 10% sucrose solutions, and spun at 100,000×g for 20 hrs at 4°C. Twelve fractions were collected from the top of the gradient, combined in pairs, and concentrated before analysis by SDS-PAGE and western blot. As lipid rafts by definition are insoluble in Triton-X-100 at 4°C [42], these microdomains and associated proteins, such as Flotillin-1 (Flot-1), floated and were over-represented in fractions 2–5, while proteins associated with the cytoplasm or detergent-soluble membranes, such as Transferrin Receptor (TFR), remained in fractions 9–12 **(** **Fig. 1D** **)**. Both Gag and Anx2 were present in fractions 3–4, indicating a proportion of both proteins localized to detergent resistant lipid raft microdomains. The increased prevalence of p55 relative to capsid (p24) in these domains is consistent with processed capsid being released from membrane-bound matrix (p17) [11].

### Bimolecular fluorescence complementation revealed that Gag and Anx2 interacted at the plasma membrane of 293T cells

To determine whether Gag and Anx2 interacted at lipid rafts, we attempted to co-immunoprecipitate Gag and Anx2 from the detergent-resistant membrane fractions but were unsuccessful, perhaps because of the relatively low protein concentrations. We thus tested our hypothesis that Gag and Anx2 interacted at lipid rafts using bimolecular fluorescence complementation (BiFC), a technique used to study protein-protein interactions in which individually inactive fragments of yellow fluorescent protein (YFP) are fused to proteins of interest. Binding of the fusion proteins when coexpressed in cells brings the YFP fragments in close proximity, allows them to fold together, and results in the formation of a fluorescing complex. Because BiFC does not require cell lysis or antibody staining, it enables the visualization of protein-protein interactions in living cells in their proper intracellular environment [65]. Furthermore, only the fraction of the expressed protein taking part in the interaction is visible, making the determination of the intracellular site of binding more precise. This technique has been used to study, in particular, membrane-associated protein complexes for which the presence of membrane is essential [65], [66] and has recently been used to study HIV Gag interactions and Gag trafficking [67], [68].

**Figure 2:**
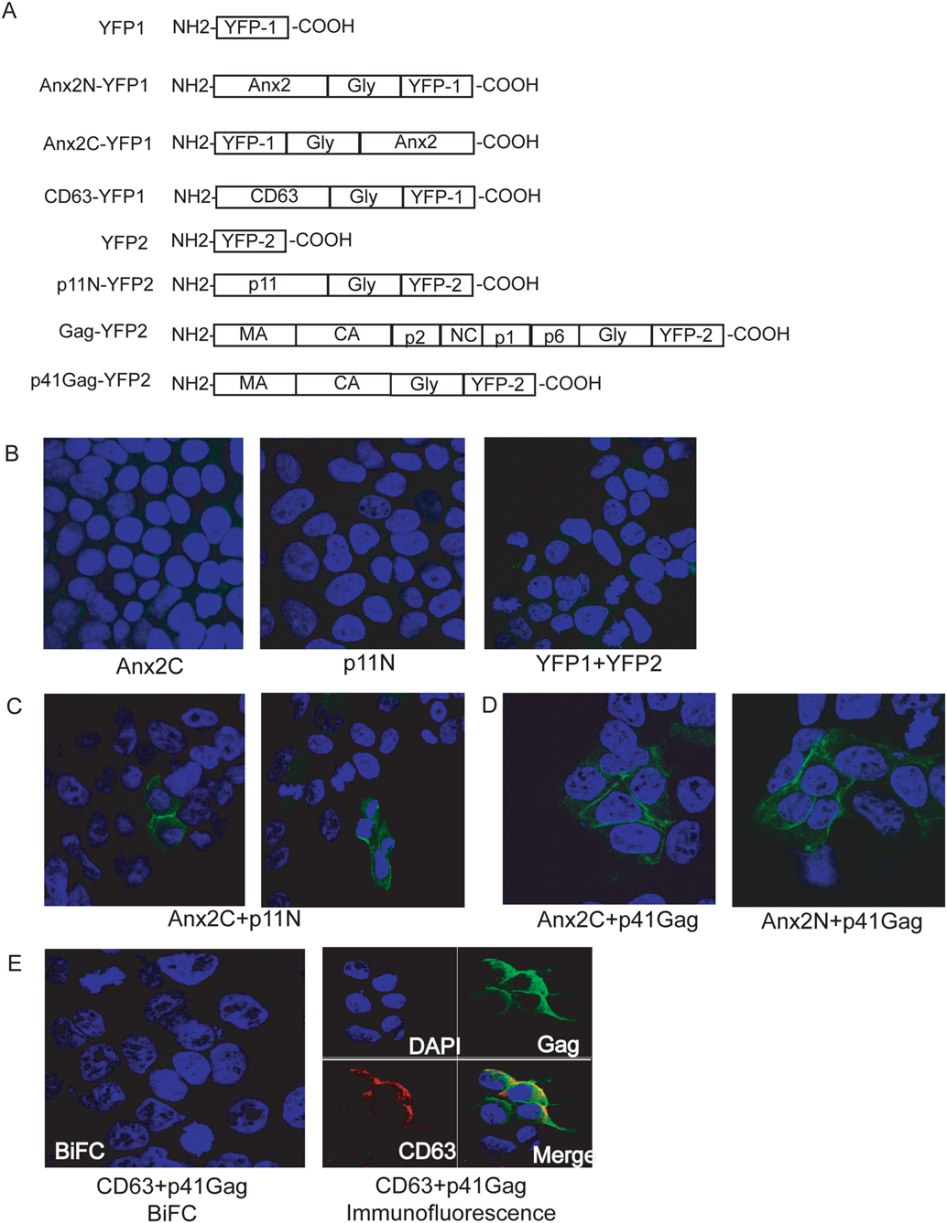
Bimolecular fluorescence complementation (BiFC) showed that Anx2 and p41^Gag^ interacted at the plasma membrane of 293T cells. 293T cells transfected with the indicated constructs were stained with DAPI and visualized by confocal microscopy 36 hours post-transfection. (A) BiFC constructs. N and C in the designation denote location at the N- or C-terminus of the construct, respectively. Gly: Glycine linker (not to scale). (B) No YFP fluorescence was detected in 293T cells transfected with single constructs or in cells cotransfected with both YFP fragments. (C) YFP fluorescence, indicating an interaction between p11 and Anx2, was detected in 293T cells cotransfected with p11 and Anx2 BiFC constructs (positive control). (D) YFP fluorescence, indicating an interaction between p41^Gag^ and Anx2, was detected at the plasma membrane of 293T cells cotransfected with p41^Gag^ and Anx2 BiFC constructs. (E) No YFP fluorescence was detected in cells cotransfected with p41^Gag^ and CD63 BiFC constructs (left panel), despite colocalized expression of CD63 and p41^Gag^ as demonstrated by immunolabeling (right panel). These data indicated CD63 and p41^Gag^ did not interact and confirmed the specificity of the fluorescence complementation between p41^Gag^ and Anx2.

We used the well-characterized interaction between Anx2 and its cellular binding partner p11 [69] to develop the BiFC assay. YFP-fragment fusion proteins were created with Anx2 fused to fragment 1 of YFP at either the C-terminus (Anx2C-FR1) or N-terminus (Anx2N-FR1) of the construct. Binding partner p11 was also fused to fragment 2 of YFP (p11N-FR2) (**Fig. 2A** **)**. Thirty-six hours after transfection with the Anx2 and/or p11 BiFC constructs, 293T cells were fixed, stained with DAPI, and visualized by confocal microscopy. Expression of either Anx2C-YFP1 or p11N-YFP2 alone did not produce YFP fluorescence **(** **Fig. 2B** **)**. Furthermore, cotransfection of constructs expressing the two YFP fragments unlinked to a protein of interest (YFP-1 and YFP-2) also failed to produce fluorescence **(** **Fig. 2B** **)**. Cotransfection of Anx2C-YFP1 with p11N-YFP2, however, resulted in fluorescence complementation, indicating a direct interaction between Anx2 and p11 **(** **Fig. 2C** **)**.

We then created two constructs with Gag fused to fragment 2 of YFP, one encoding full-length Gag and another encoding the N-terminal matrix-capsid region of Gag (p41^Gag^) **(** **Fig. 2A** **)**. Cotransfection of p41^Gag^ and Anx2C resulted in fluorescence complementation as demonstrated in **Fig. 2D**. There was no difference in signal intensity or localization when Anx2N was used instead of Anx2C, and Anx2C was therefore used for all further experiments. While both Gag constructs produced fluorescence complementation when cotransfected with Anx2, the signal resulting from p41^Gag^ was greater than that from full-length Gag (not shown). Complementation between p41^Gag^ and Anx2C indicated that the Anx2 binding site on Gag is located in the matrix and/or capsid regions, and the reduced fluorescence observed with full-length Gag is likely due to the greater distance between YFP fragments in the Anx2-Gag complex. The YFP fluorescence was detected at the plasma membrane of p41^Gag^ and Anx2C cotransfected cells **(** **Fig. 2D** **)**, indicating that the Anx2-p41^Gag^ interaction occurred at the plasma membrane, the site of virion assembly and budding in 293T cells [70]–[73]. There was no signal attributable to binding within intracellular organelles or vacuoles.

To ensure that the fluorescence complementation between Anx2C and p41^Gag^ represented a direct interaction between the two proteins and not mere close proximity, we prepared a CD63-YFP fragment 1 construct as a negative control **(** **Fig. 2A** **)**. In 293T cells, CD63 is expressed at the plasma membrane in addition to intracellular compartments and is incorporated into released HIV-1 virions [[74] and data not shown)] and is therefore present near the site of viral assembly. Cotransfection of CD63 and p41^Gag^ produced no fluorescence despite expression of both CD63 and p41 in similar regions of the cell as indicated by immunolabeling of the proteins and examination by confocal microscopy **(** **Fig. 2E** **)**. This result confirmed the specificity of the Anx2-p41^Gag^ interaction.

### Anx2 and Gag interacted at GM1-containing lipid rafts in the plasma membrane of 293T cells

**Figure 3:**
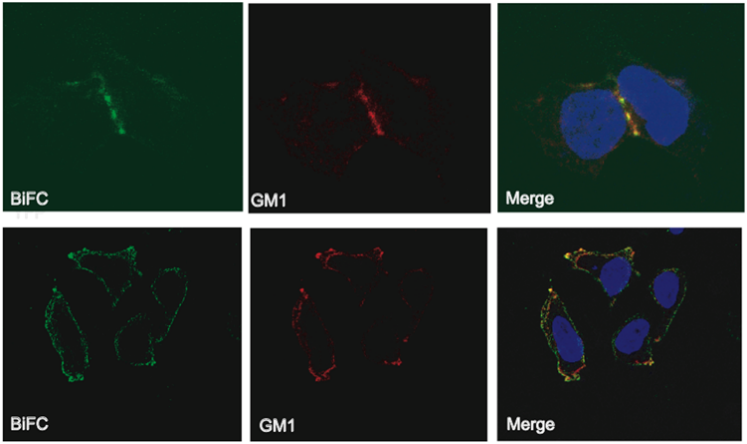
Anx2 and Gag interacted at GM-1-containing lipid rafts in the plasma membrane of 293T cells. 293T cells cotransfected with p41^Gag^ and Anx2 BiFC constructs were fixed and incubated with Alexa Fluor® 594 conjugated cholera toxin B (CTB) to label the lipid raft component ganglioside M1 (GM1) followed by crosslinking of the raft regions with anti-CTB antibody. YFP colocalized with CTB-containing patching, indicating that p41^Gag^ and Anx2 interacted at GM1-containing lipid rafts.

HIV-1 preferentially assembles at and buds from lipid raft membrane microdomains [16]–[19]. To determine whether the p41^Gag^-Anx2 interaction occurred at these regions of the plasma membrane, 293T cells transfected with the BiFC constructs were incubated with Alexa Fluor® 594-conjugated cholera toxin B (CTB), which binds to the raft component ganglioside M1 (GM1) [75], [76]. The CTB-labeled rafts were then crosslinked with anti-CTB antibody in order to create distinct raft patches on the plasma membrane [77] and visualized by confocal microscopy. YFP fluorescence colocated with the GM1-containing membrane patches, indicating that the Anx2-p41^Gag^ interaction occurred on GM1-containing lipid raft regions of the plasma membrane **(** **Fig. 3** **)** and providing further support that Gag and Anx2 interact at the site of virion assembly and budding.

### The Anx2-Gag interaction was dependent on the intracellular location and levels of PtdIns(4,5)P_2_

Along with lipid raft components, the phospholipid PtdIns(4,5)P_2_, plays an important role in the targeting and binding of Gag to the plasma membrane [21]. Whereas in normal HeLa cells Gag is directed to the plasma membrane, redirection of PtdIns(4,5)P_2_ from the plasma membrane to internal PtdIns(4,5)P_2_-rich endosomal structures also redirects Gag and viral budding to these structures [20]. Depletion of PtdIns(4,5)P_2_ altogether also causes failure of Gag trafficking to the plasma membrane and instead accumulation of Gag in late endosomes [20]. Both manipulations of PtdIns(4,5)P_2_ reduce viral production [20].

**Figure 4:**
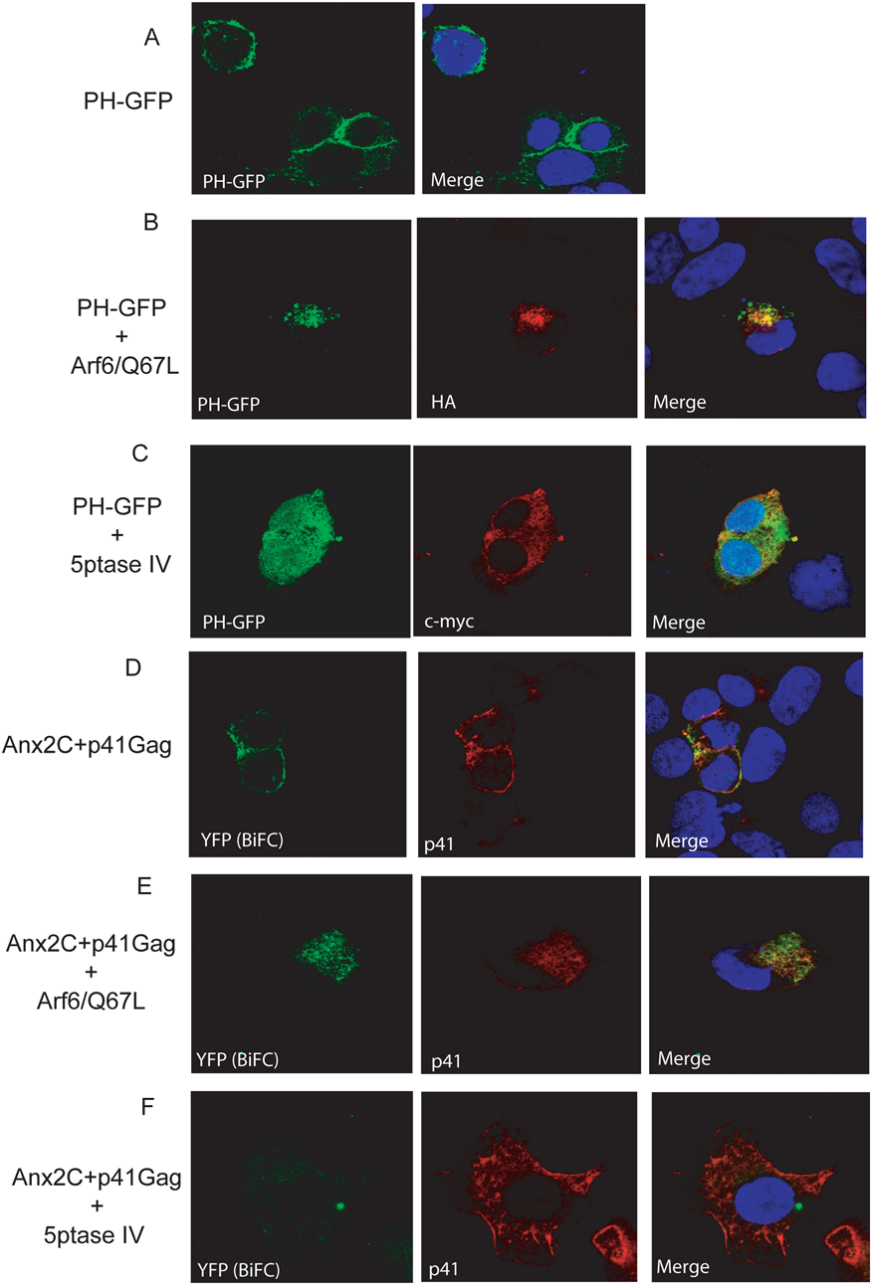
The Anx2-p41^Gag^ interaction occurred at PtdIns(4,5)P_2_-enriched membrane domains and depended on intracellular levels of PtdIns(4,5)P_2_. 293T cells transfected with the indicated constructs were immunostained for protein (p41) or tag (HA, c-myc), labeled with DAPI and visualized by confocal microscopy 36 hours post-transfection. (A) PH_PLCδ_-GFP labeled PtdIns(4,5)P_2_ at the plasma membrane of 293T cells. (B) Arf6/Q67L redirected PtdIns(4,5)P_2_ and thus PH_PLCδ_-GFP to intracellular membranes. Cells expressing Arf6/Q67L were identified by immunolabeling for the HA tag (middle panel). (C) 5ptase IV depletes PtdIns(4,5)P_2_ and causes PH_PLCδ_-GFP to be diffusely distributed throughout the cytoplasm and nucleus. Cells expressing 5ptase IV were identified by immunolabeling for the c-myc tag (middle panel). (D) In 293T cells with normal PtdIns(4,5)P_2_ expression, the Anx2-p41^Gag^ interaction, as denoted by YFP fluorescence complementation, occurred at the plasma membrane where PtdIns(4,5)P_2_ is located. (E) The Anx2-p41^Gag^ interaction was redirected to PtdIns(4,5)P_2_ –enriched intracellular membranes when 293T cells expressed Arf6/Q67L. (F) Depletion of with 5ptase IV inhibited the Anx2-p41^Gag^ interaction as indicated by reduced YFP fluorescence complementation. Reduced binding between Anx2 and p41^Gag^ is not due to reduced p41^Gag^ expression, as determined by immunolabeling of p41^Gag^ (middle panel).

To determine whether the Anx2-Gag interaction occurred on PtdIns(4,5)P_2_-containing membrane domains, we altered the levels and intracellular locations of PtdIns(4,5)P_2_ and investigated the effects of these manipulations on fluorescence complementation between Anx2 and p41^Gag^. The results described below were the predominant effects observed in multiple fields of cells. To visualize PtdIns(4,5)P_2_, we used a construct expressing GFP fused to the pleckstrin homology (PH) domain of phospholipase C-δ (PH_PLCδ_-GFP) that has been shown to bind to PtdIns(4,5)P_2_ [78]. When transfected alone into 293T cells, PH_PLCδ_-GFP localized primarily to the plasma membrane as previously demonstrated **(** **Fig. 4A** **)** [20], [78]. To redistribute PtdIns(4,5)P_2_ away from the plasma membrane, we cotransfected 293T cells with PH_PLCδ_-GFP and HA-tagged ADP-ribosylation factor 6/Q67L (Arf6/Q67L), a constitutively active form of Arf6 which causes intracellular accumulation of PtdIns(4,5)P_2_-enriched endosomal structures [79], [80] to which Gag [20] and Anx2 [81] are directed. Cotransfection of Arf6/Q67L with PH_PLCδ_-GFP redistributed GFP expression away from the plasma membrane and into intracellular formations, indicating redirection of PtdIns(4,5)P_2_ **(** **Fig. 4B** **)**. When co-transfected with myc-tagged phosphoinositide-specific inositol polyphosphate 5-phosphatase IV (5ptase IV), an enzyme that depletes PtdIns(4,5)P_2_ [82], GFP became diffusely distributed throughout the cell, indicating depletion of PtdIns(4,5)P_2_ from the plasma membrane **(** **Fig. 4C** **)**; this effect has been previously observed in HeLa cells [20].

We next tested the effect of these manipulations of PtdIns(4,5)P_2_ on BiFC between Anx2 and p41^Gag^ in 293T cells. As described above, transfection of Anx2 and p41^Gag^ resulted in YFP fluorescence at the plasma membrane, indicating that the site of viral assembly is the site of interaction between these two proteins **(** **Fig. 4D** **)**. Cotransfection of the BiFC constructs with Arf6/Q67L redirected YFP from the plasma membrane to intracellular compartments, indicating that the Anx2-Gag complex was redirected to the PtdIns(4,5)P_2_-containing endosomal structures **(** **Fig. 4E** **)**. When Anx2 and p41^Gag^ were cotransfected with 5ptase IV, YFP was no longer detected, indicating that the absence of PtdIns(4,5)P_2_ inhibited the interaction between the two proteins **(** **Fig. 4F** **)**. This failure of fluorescence complementation is despite expression of p41^Gag^ that was comparable to cells in which 5ptase IV is not expressed as indicated by immunolabeling of Gag **(** **Figs. 4D, F** **middle panels)**. These data indicated not only that the Anx2-Gag interaction occurred preferentially on PtdIns(4,5)P_2_-enriched membranes, but also that the presence of PtdIns(4,5)P_2_ was necessary for this interaction to occur efficiently.

Our experiments demonstrating that Gag and Anx2 interacted at the PtdIns(4,5)P_2_ enriched lipid raft membrane domains at which viral assembly occurs in 293T cells support a role for this protein in HIV-1 assembly and budding. A functional role for Anx2 was also suggested by our previous loss-of-function studies showing that siRNA-mediated downregulation of Anx2 in macrophages led to decreased viral replication, aberrant Gag processing, and failure of mature virions to accumulate in intracellular vesicles [9]. To identify a gain-of-function role for Anx2, we compared Gag processing and viral production in 293T cells co-transfected with HIV-1_YU2_ and Anx2 to those co-transfected with HIV-1_YU2_ and empty vector.

### Anx2 gain-of-function increased Gag processing and HIV-1 production in 293T cells

**Figure 5:**
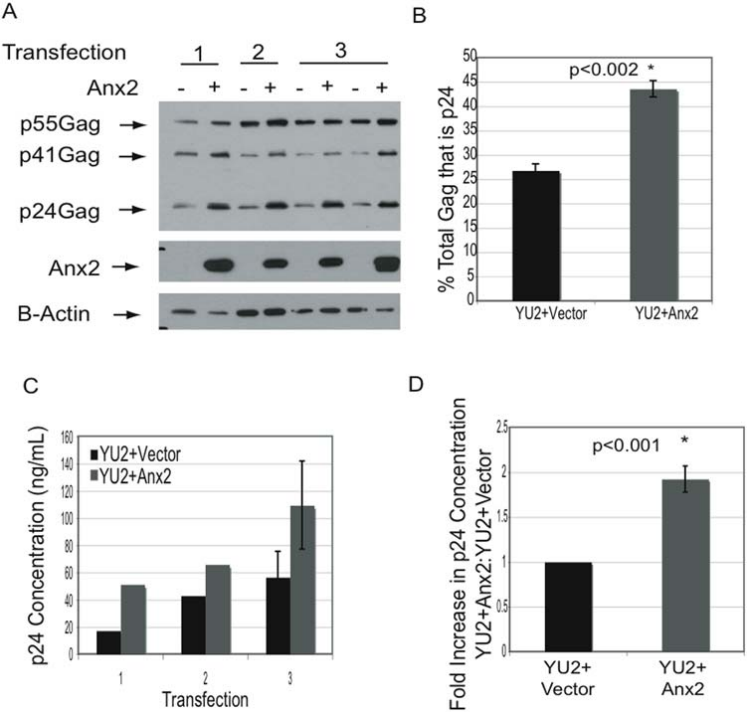
Anx2 gain-of-function in 293T cells increased Gag processing and viral production. (A) 293T cells were cotransfected with HIV-1_YU2_ and either empty vector or Anx2. At 36–48 hrs after transfection, the cells were lysed in 0.5% Triton-X. Lysates from 3 independent transfections were analyzed by SDS-PAGE and western blot with HIV-Ig, anti-Anx2, and anti-β-actin. Anx2 increased processing of p55^Gag^ into mature capsid (p24^Gag^). (B) Quantification of the western blot in (A) with NIH Image J. Anx2 increased the percent of total Gag processed into mature capsid (p24^Gag^) (p<0.002) using a correlated samples two-tailed student's t-test. Error bars represent standard deviations. (C) The p24^Gag^ concentration of the lysates shown in (A) was determined by ELISA. Anx2 increased the mature capsid concentration as suggested by the western blot analysis in (A). (D) Lysates from 19 different transfections of HIV-1_YU2_ with either empty vector or Anx2 were prepared as in (A). p24^Gag^ concentrations of the lysates were determined by ELISA. The average ratio of the experimental values (HIV-1_YU2_+Anx 2) to the control value (HIV-1_YU2_+vector) are shown on the bar graph. The error bars represent standard errors of the means. Statistical analysis was performed using paired two-tail student's t test. Anx2 increased the intracellular concentration of mature capsid.

We noted that in many co-transfection experiments Anx2 expression appeared to enhance the processing of Gag into mature capsid (p24^Gag^), an event associated with maturation of assembled virions. To quantify this effect, we analyzed cell lysates from three independent transfections in which this enhancement was seen. Lysates were subjected to SDS-PAGE followed by western blotting with HIV Immunoglobulin and antibody to β-actin **(** **Fig. 5A** **)**. When normalized to β-actin levels, Anx2 increased total Gag levels in each transfection, but when averaged over all 3 experiments the increase was not statistically significant (p = 0.096) However, Anx2 did increase the percent of total Gag that was processed into mature capsid (p24) from an average of 26.8% to 43.6%, an effect that was highly statistically significant (p<0.002) **(** **Fig. 5B** **)**. Measurement of intracellular p24^Gag^ concentration in these lysates by ELISA confirmed that Anx2 increased intracellular capsid levels **(** **Fig. 5C** **).** The increase in Gag processing was not observed in every transfection, so to better quantify the overall effects of Anx2 on intracellular capsid levels, lysates from 19 independent co-transfections of 293T cells with HIV-1_YU2_ and Anx2 were analyzed for p24^Gag^ concentration using ELISA. Anx2 increased the intracellular p24^Gag^ concentration in 15/19 experiments; the average increase of all experiments was 90.2% (**Fig. 5D**).

**Figure 6:**
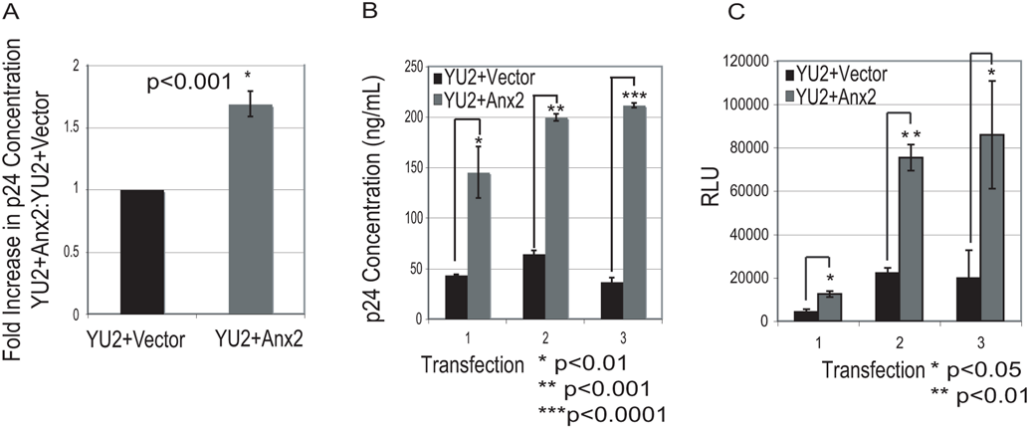
Anx2 gain-of-function in 293T cells increased the production of infectious HIV-1. (A) 293T cells were cotransfected with HIV-1_YU2_ and either empty vector or Anx2. At 36–48 hrs after transfection, the supernatants were collected and the p24^Gag^ concentrations were determined by ELISA. The average ratios of the experimental values (HIV-1_YU2_+Anx 2) to the control values (HIV-1_YU2_+vector) are shown on the bar graph. Data represent 46 different transfections; error bars represent standard errors of the means. Statistical analysis was performed using paired two-tail student's t test. Anx2 increased HIV-1_YU2_ production by 293T cells. (B) p24^Gag^ concentrations in the supernatants from three independent transfections of 293T cells with either HIV-1_YU2_+Vector or HIV-1_YU2_+Anx2 were measured by ELISA. Anx2 increased viral production in each transfection. Error bars represent standard deviations. Statistical analysis was performed using unpaired two-tail student's t test. (C) Quantification of infectious virus in the supernatants shown in (B) using TZM-bl cells expressing the luciferase gene under the control of the HIV-1 promoter. TZM-bl were infected with equal volumes of supernatants from both control and Anx2-expressing 293T cells and luciferase expression in relative light units (RLU) was determined after 24 hours and taken as a measure of the amount of infectious virus present. More infectious virus was present in the supernatants of Anx2-expressing 293T cells in comparison with control cells in each of the three transfections. Error bars represent standard deviations. Statistical analysis was performed using unpaired two-tail student's t test.

In previous experiments, depletion of Anx2 in HIV-1 infected macrophages not only impaired Gag processing but also reduced virus production [9]. To determine whether Anx2 expression in 293T cells could have the opposite effect (i.e. increased viral production), we measured p24^Gag^ concentration in the supernatants of the cells from 47 separate co-transfections of HIV-1_YU2_ with either Anx2 or empty vector at 36 hr post-transfection. Anx2 overexpression was associated with increased p24^Gag^ release into the supernatant by an average of 68.8% (**Fig. 6A**). As with Gag processing, however, there was experimental variability in the effects of Anx2 on viral production, likely due to differences in transfection efficiency and cell density between transfections. To ensure that the Anx2-mediated increase in mature capsid represented an increase in the production of infectious virus, supernatants from three independent transfections demonstrating this increase were used to infect TZM-bl cells, which express the luciferase gene under control of the HIV-1 promoter and which have been used previously to quantify virus infectivity [83]–[85]. The p24^Gag^ concentrations in cell supernatants from these three transfections are shown in **Fig. 6B**; Anx2 significantly increased the concentration of mature capsid in each transfection. To quantify infectious virus, equal volumes of these supernatants were applied to TZM-bl cells for 24 hours. The amount of infected cells in each condition was then quantified by analyzing luciferase expression **(** **Fig. 6C** **)**. Consistent with the measured mature capsid concentrations, supernatants from cells expressing Anx2 contained significantly more infectious HIV-1 than control cells in each of the three transfections. These data indicated Anx2 gain-of-function in 293T cells led to enhanced Gag processing and increased infectious viral production and thus support a functional role for Anx2 in HIV-1 assembly and budding.

## Discussion

Here we present further evidence that Anx2 plays a role in HIV-1 assembly and budding by characterizing the intracellular site of the Anx2-Gag interaction and by investigating the effects of Anx2 gain-of-function on viral replication in 293T cells. Bimolecular fluorescence complementation demonstrated that Anx2 and Gag interacted at GM1-containing lipid rafts in the plasma membrane, the membrane microdomains at which HIV assembles in these cells [15], [18], [19], [70]–[73]. Efficient formation of the Anx2-Gag complex depended on the presence of PtdIns(4,5)P_2_, a membrane phospholipid that targets Gag to the plasma membrane and facilitates Gag-membrane binding [21]–[23], [86]. Redirection of PtdIns(4,5)P_2_ to intracellular membranes – a manipulation that also redirects viral assembly [86] – also redirected the complex to the cytoplasm. The presence of Anx2 at the HIV-1 assembly site was confirmed in the context of whole virus replication in 293T cells, as Anx2 was present with Gag in detergent-resistant membrane fractions containing lipid rafts and in the released virions. Finally, Anx2 gain-of-function in 293T cells led to enhanced Gag processing and increased viral production, findings that complement our previous studies showing that knockdown of Anx2 in MDMs led to impaired Gag processing and decreased viral production [9].

Based on the known functions of Anx2, we propose that the most likely mechanism behind our results is Anx2-mediated creation and/or stabilization of the PtdIns(4,5)P_2_-containing lipid raft membrane domains from which the virus preferentially buds [16]–[21]. In this model, Anx2-Gag binding would function to recruit Gag to these domains. PtdIns(4,5)P_2_ interacts directly with hydrophobic residues in the matrix domain of Gag and triggers exposure of the N-terminal myristic acid [21], an event that is important for membrane-binding [62], [64], [87], [88]. A recent *in vitro* study confirmed that efficient binding of Gag to liposomes requires the presence of PtdIns(4,5)P_2_ [22]. After initial membrane binding, Gag becomes associated with the highly ordered membrane microdomains rich in sphingolipids and cholesterol known as lipid rafts [19]. This localization may be facilitated by the Gag-PtdIns(4,5)P_2_ interaction, as the unsaturated 2′ side chain of the phospholipids leaves the lipid bilayer to bind matrix, leaving the saturated 1′ fatty acid side chain [21], [23]. Other studies have suggested that lipid rafts may serve as platforms that enable the higher order Gag multimerization that occurs after initial membrane binding [19], [24], [63], [89], [90].

Anx2 can laterally aggregate and stabilize cholesterol-rich microdomains [39], [40]. Furthermore, Anx2 binds directly to PtdIns(4,5)P_2_ [81], [91] and can induce the formation of stable PtdIns(4,5)P_2_ clusters within membranes in a cholesterol-dependent manner [45]. At intracellular membranes in macrophages and at the plasma membrane of 293T cells, Anx2 may thus help to create and/or stabilize domains that enhance initial Gag-membrane binding and facilitate Gag-Gag multimerization and increase viral production. This mechanism would explain increased Gag processing, as enhanced lipid raft-binding and Gag multimerization would in turn facilitate dimerization of Gag-Pol precursors within the assembling virion [92] and thus activation of the viral protease. As noted, siRNA-mediated depletion of Anx2 in HIV-1 infected macrophages not only reduced overall virion production but also induced defects in Gag processing that were associated with reduced infectivity of released virions, effects that we hypothesized resulted from destabilization of lipid rafts in the absence of Anx2 [9].

The localization of Anx2-Gag complexes to GM1 and PtdIns(4,5)P_2_-containing membrane domains, as well as the dependence of the interaction on the levels and location of PtdIns(4,5)P_2_, are consistent with the scenario in which Anx2-mediated stabilization of such domains is important for Anx2-Gag binding and HIV-1 assembly. Of note, in HIV-1 infected MDMs Anx2 and Gag collocated with CD63 positive internal membranes, in contrast to the plasma membrane localization of the Anx2-Gag BiFC complexes in 293T cells. Importantly, the membranes at which the interaction occurs represent the site of viral assembly in each cell type [48]–[50], [70]–[73], [93]. In both MDMs [9] and transfected 293Ts (not shown), Anx2 expression was diffusely distributed and not confined to areas of collocation with Gag. Our data do not distinguish between the two most likely explanations for the different sites of the Gag-Anx2 interaction: *first*, that the ability of Anx2 to perform its function in viral assembly is spatially regulated by cell-specific factors in endosomal membranes in MDMs and in the plasma membrane in 293T cells, leading to Gag recruitment to those sites; or *second*, that Anx2 performs its function throughout each cell but Gag targeting is mediated by another cell-specific molecule or mechanism. Cell-specific distribution of PtdIns(4,5)P_2_ may be one such mechanism, as its redistribution from the plasma membrane redirected both viral assembly [20] and the Anx2-Gag interaction to PtdIns(4,5)P_2_-enriched internal membranes. We are currently performing studies to determine the relationship between PtdIns(4,5)P_2_ localization and Anx2 function in MDMs.

There are several other potential, though not mutually exclusive, explanations for the increase in viral production seen with Anx2. For example, binding between Anx2 and Gag could induce conformational changes in Gag, possibly triggering the myristyl switch that enhances Gag-membrane binding. Anx2 can also promote the aggregation and fusion of membrane-bound structures such as secretory granules with the plasma membrane and endosomes with each other [94]–[99]. The latter function is thought to be relevant in CMV infection, as Anx2 can enhance the fusion of CMV with phospholipid-containing membranes [100], [101]. Anx2 might similarly facilitate this process for HIV, which also requires fusion events for budding. Anx2 is an actin-binding protein [102], [103] with the ability to regulate actin polymerization [104] and may act as a membrane-cytoskeletal scaffolding protein [105]. Anx2 could thus facilitate HIV production by mediating Gag-actin interactions and Gag trafficking. Finally, as Anx2 is a multifunctional protein, overexpression could have a general effect on cellular processes and only indirectly increase viral assembly. Cotransfection of Anx2, however, did not produce evident structural abnormalities by EM (data not shown) or change the expression of cellular proteins such as GAPDH or B-actin **(** **Figs. 1A** **,** **5A** **)**, arguing for a relatively specific effect of Anx2.

Future experiments will more precisely determine the mechanism by which Anx2 facilitates Gag-mediated assembly, budding, processing and release. Ultimately, we hope to gain sufficient understanding of this mechanism to determine the function of Anx2 in HIV-1 replication in macrophages. As the host proteins that regulate HIV replication are likely to be different in T cells and macrophages [106], such an understanding will be crucial in the eventual development of therapies to counteract the roles of HIV-1 infected macrophages in viral transmission, reservoir formation, and neuropathogenesis.

## Materials and Methods

### Plasmids and cell transfection

We received several plasmids as gifts from other laboratories: the HIV-1_YU2_ proviral genome (RF-1) from Dr. M. Malim, King's College, London; PH_PLCδ_-GFP from Dr. T. Balla, National Institutes of Health [78]; 5ptase IV from Dr. P.W. Majerus, Washington University School of Medicine, St. Louis, MO [82]; and Arf6/Q67L form Dr. J.G. Donaldson, National Institutes of Health. Anx2 was cloned into the pcDNA3.1(+) vector (Invitrogen) under control of the human CMV promoter. For BiFC, the coding sequence for the N-terminal 160 residue fragment of YFP (FR1) along with a linker segment of 12 glycines was cloned into the pcDNA3.1(−) vector attached to either the N- or C-terminus of Anx2. To construct p41^Gag^-FR2, the matrix and capsid domains of p55^Gag^ were amplified from the codon-optimized pGagEGFP plasmid, a gift from Dr. M. Resh, Memorial Sloan-Kettering Cancer Center. Four constitutive transport elements (CTE; a gift from Dr. M. Malim, King's College, London; [107]) were cloned into the 3′UTR of p41Opt. The p41^Gag^ segment and the C-terminal 82 residues of YFP (FR2) along with a linker segment of 12 glycines were cloned into the pcDNA3.1(−) vector.

293T cells were transfected using the calcium-phosphate method (Promega ProFection kit). For studies of the effect of Anx2 on HIV replication, 1 ug YU-2 plasmid was co-transfected with 3 ug of Anx2 or pcDNA 3.1. For BiFC, Anx2-FR1 and p41^Gag^-FR2 were co-transfected in the ratio of 1 ug Anx2 or CD63: 2 ug p41^Gag^ or Gag along with 1 ug empty vector, 5ptase IV, or Arf6/Q67L.

### Bimolecular fluorescence complementation (BiFC) and Immunofluorescence

Thirty-six hours after transfection, 293T cells were fixed in 4% paraformaldehyde in PBS and permeabilized with 0.1% Triton-X. Gag-YFP2 was immunolabeled with mouse anti-GFP (Roche Applied Science, Germany), 5ptase IV with rabbit anti-c-Myc (A-14) antibody (Santa Cruz), and Arf6/Q67L with rabbit anti-HA-probe (Y-11; Santa Cruz). Cells were then incubated with Alexa Fluor® 488 chicken anti-mouse or anti-rabbit IgG (Invitrogen), stained with DAPI (4′,6′-diamidino-2-phenylindole; Invitrogen) and coverslipped with ProLong® Gold antifade reagent (Invitrogen). For collocation with GM1, cells were stained with recombinant cholera toxin B (CTB) conjugated to Alexa Fluor® 594 after fixation and CTB was then aggregated using anti-CTB antibody (Vybrant® Lipid Raft Labeling kit, Invitrogen). Fluorescence in the cells was analyzed using a Zeiss LSM 510 confocal microscope. Images were taken with a LSM 510 camera and processed with Adobe Photoshop CS.

### Membrane flotation

Membrane flotation was performed as described previously [62]–[64]. Thirty-six hours post-transfection, 293T cells were lysed in Tris-EDTA (TE) buffer (10 mM Tris-HCl pH 7.5 with 1 mM EDTA) containing 1% Triton X-100, 6% sucrose, and protease inhibitors. Post-nuclear supernatants (PNS) were obtained by low-speed centrifugation and 250 µL were adjusted to 73.3% sucrose with 1.25 mL 85.5% sucrose in TE. The lysates were overlaid with 7 mL 65% sucrose in TE and 3.25 mL 10% sucrose in TE before ultracentrifugation at 100,000×g for 18–24 hrs at 4°C. Twelve 1 mL fractions were collected from the top, and 150 µL fractions were combined in pairs and proteins concentrated using the Pierce SDS-PAGE Sample Prep Kit according to the manufacturer's protocol. Fractions were analyzed by SDS-PAGE and western blot with rabbit polyclonal antiserum against HIV-1_SF2_ p24^Gag^ (AIDS Research and Reference Reagent Program, Division of AIDS, NIAID, NIH), mouse anti-Anx2 (BD Biosciences, New Jersey), mouse anti-Human Transferrin Receptor (Zymed, Invitrogen, Carlsbad, CA), and mouse anti-Flotillin-1 (BD Biosciences).

### Virus precipitation and analysis

Peripheral blood mononuclear cells derived from whole blood by the University of Pennsylvania Center CFAR Immunology core were plated (3.2 million cells/well) in six-well plates and differentiated into macrophages for seven days. 293T cell supernatants containing HIV-1_YU2_ (200 ng p24^Gag^ per well as measured by p24^Gag^ ELISA) were applied to the monocyte-derived macrophages (MDMs) for 48 hours, and infected MDM supernatants were collected 11 days after infection. Cellular material was pelleted by centrifuging the supernatants for 1 min at 16,100×g and then 1 mL of each clarified supernatant was passed through 0.45 µm filters to further remove any cellular components. Virus pellets were obtained by ultracentrifugation of the filtered supernatants and then analyzed by SDS-PAGE and western blotting with rabbit polyclonal antiserum against HIV-1_SF2_ p24^Gag^ (AIDS Research and Reference Reagent Program, Division of AIDS, NIAID, NIH), mouse monoclonal anti-Anx2 antibody (BD Biosciences, New Jersey), and rabbit polyclonal anti-GAPDH antibody (Abcam, Cambridge, MA). Virus pellets from transfected 293T cells were prepared and analyzed in the same manner.

### Virus immunoprecipitation from cell supernatants

Supernatants from 293T cells transfected with Anx2 alone or HIV-1_YU2_+Anx2 were collected 36 hours post-transfection. Before immunoprecipitation, the cellular debris was pelleted by centrifugation for 1 min at 16000×g. 900 µL of each supernatant was then added to 100 µL of PBS only or to 100 uL 5% Triton-X in PBS to lyse the virions. The supernatants were pre-cleared with 50 µL recombinant protein G agarose (Invitrogen, Carlsbad, CA) at 4°C for one hour before incubation with 5 µL HIV Immunoglobulin (HIV-IG™ from AIDS Research and Reference Reagent Program, Division of AIDS, NIAID, NIH) overnight at 4°C. Fifty µL of fresh protein G beads were then added to the supernatant-antibody mixture and incubated for 2 hr at 4°C. The beads were washed twice in 0.5% Triton-X in PBS, once in PBS, resuspended in 50 µL 2× SDS buffer and boiled for 5 min. Equal volumes of immunoprecipitates were subjected to SDS-PAGE on 8–16% Tris-Glycine polyacrylamide gels (Lonza, Switzerland) and western blotting with mouse monoclonal antibody against HIV-1 p24^Gag^ (#24-2, AIDS Research and Reference Reagent Program, Division of AIDS, NIAID, NIH) and mouse monoclonal anti-Anx2 antibody (BD Biosciences, New Jersey).

### Western blotting for Anx2 expression and Gag Processing

293T cells at 36 hours post-transfection and MDMs at 10–14 days after infection with HIV-1_YU2_ were lysed in 0.5% Triton-X in PBS with protease inhibitors (Complete, Roche Applied Science, Germany) and protein concentrations were determined using the Bio-Rad *DC* Protein Assay kit. Equal micrograms of each sample were analyzed by SDS-PAGE and western blot. Antibodies used for protein detection were HIV Immunoglobulin (HIV-IG™ from AIDS Research and Reference Reagent Program, Division of AIDS, NIAID, NIH), mouse anti-Anx2 (BD Biosciences, New Jersey), mouse monoclonal against beta actin (Abcam, Cambridge, MA), and rabbit polyclonal against GAPDH (Abcam, Cambridge, MA). NIH Image J was used to quantify bands. Statistical analysis was performed using a two-tailed paired student's t test.

### p24^Gag^ protein measurement

36 hours post-transfection of 293T cells, supernatants were collected and the cells were lysed in 0.5% Triton-X in PBS supplemented with protease inhibitors (Complete; Roche Applied Science, Germany). The Gag concentration in the supernatants and cell lysates was measured in our laboratory using the p24^Gag^ enzyme-linked immunosorbent assay (ELISA) as per the manufacturer's instructions (Perkin-Elmer; NEK050) or by the University of Pennsylvania Center for AIDS Research (CFAR) Virus and Molecular Core. The effect of Anx2 overexpression on Gag concentration was calculated as the ratio of the experimental values (HIV-1_YU2_+Anx2) over the control values (HIV-1_YU2_+vector) with control values set to one. Statistical analysis was performed on the ratios using a paired two-tailed student's t-test. Error bars represent standard errors of the mean.

### Quantification of infectious virus in 293T supernatants

TZM-bl cells are CD4- and CCR5-expressing HeLa cells that contain the luciferase and β-galactosidase genes under the control of the HIV-1 promoter (obtained from the NIH AIDS Research and Reference Reagent Program, Division of AIDS, NIAID, NIH: from Dr. John C. Kappes, Dr. Xiaoyun Wu, and Tranzyme Inc.) TZM-bl cells in 96-well plates were incubated with 5 uL of virus-containing supernatant from HIV-1_YU2_+Vector and HIV-1_YU2_+Anx2 transfected 293T cells. Supernatants from three independent transfections were used. After 24 hours of incubation, the media was removed and the cells were lysed by exposure to 25 uL 1× Reporter Lysis Buffer (Promega, Madison, Wisconsin) followed by one freeze-thaw cycle. One-hundred microliters of Luciferase Assay Substrate (Promega) was added to each well and the relative light units (RLU) were measured using a Packard LumiCount luminometer and Packard PlateReader 3.0 software. Statistical analysis was performed using an unpaired two-tailed student's t-test.
